# Interdigital concept in photonic sensors based on an array of lossy mode resonances

**DOI:** 10.1038/s41598-021-92765-0

**Published:** 2021-06-24

**Authors:** Ismel Dominguez, Ignacio Del Villar, Omar Fuentes, Jesus M. Corres, Ignacio R. Matias

**Affiliations:** 1 Institute of Smart Cities, Public University of Navarre, 31006 Pamplona, Spain; 2 Department of Telecommunications and Electronics, Pinar del Río University, CP 20100 Pinar del Río, Cuba; 3 Department of Electrical, Electronic and Communications Engineering, Public University of Navarre, 31006 Pamplona, Spain

**Keywords:** Optical sensors, Optoelectronic devices and components

## Abstract

Multi-parameter detection is key in the domain of sensors. Here it is demonstrated that an indium tin oxide (ITO) nanocoating can be used to generate multiple lossy mode resonances (LMRs) in the optical spectrum. To achieve this, a nanocoating with a gradient in thickness is generated on the surface of a planar waveguide, permitting broadening of the LMR because the position of an LMR in the optical spectrum is directly related to the nanocoating thickness. The nanocoating with a gradient in thickness contributes multiple LMRs, each one centred at a different wavelength. With a further etching or deposition using a mask, a pattern of deposited and non-deposited regions can be created, resulting in isolation of the LMRs by preventing LMR overlap. This enables tracking of each central wavelength separately, which can be tuned through control of the gradient or nanocoating pattern. The array of LMR-based sensors is a photonics analogue to the interdigital concept in electronics, enabling multiple resonances to be used for multiparameter sensing.

## Introduction

Interdigital electrodes are a broadly-used type of periodic electrode structure^1^ important in various applications including telecommunications, non-destructive testing, optical photodetectors, and sensors. They are employed within biomedical, environmental, and industrial sensors^2^. Interdigital electrodes are most widely used in capacitive sensors^3,4^, though other examples include inductive, dielectric, piezoacoustic, chemical, and microelectromechanical (MEMS) interdigital sensors and transducers^1^. Despite their success, interdigital electrodes are not implemented in electronic sensors. Capacitive interdigital sensors are typically optimised for high effective capacitance per unit area. In this work, we optimise instead the density of sensor units. We demonstrate the generation of an array of lossy mode resonances (LMRs) at different wavelengths in the optical spectrum by electrodes of differential thickness. The resonance wavelength of each LMR is tuned separately by modifying the refractive index of the medium surrounding the sensor. We anticipate that this concept could be expanded to the application of a voltage to these resonance-generating electrodes, either in a parallel or serial configuration, and interrogating sensor units together or independently. Here we introduce the concept and implementation of photonic interdigital sensors using generation of LMRs by deposition of gradient-thickness thin films on planar waveguides.

Lossy mode resonances (LMRs)^5,6^, also known as guided mode resonances^7^, are based on the generation of attenuation bands in the optical spectrum when modes guided in a substrate experience a transition to guidance in a thin film deposited on the substrate under the following conditions: the real part of the thin-film permittivity must be positive, and it must be higher in magnitude than both its own imaginary part and the material surrounding the thin film^7,8^. For this reason, they are typically generated with polymers and metallic oxides^8,9^. The incidence angle for excitation if LMRs approaches 90º^10^, which is an important factor in their success in thin-film coated optical fibres, where the propagation of light is parallel to the thin film.

In addition, it is possible to separate the transverse electric (TE) and the transverse magnetic (TM) component of the LMR when non-cylindrically symmetric structures such as D-shaped fibres are used^11,12^, which permits to track with more accuracy the resonance wavelength. However, more recently, planar waveguides such as a glass slides or coverslips^13^ have become an alternative to D-shaped fibres due to their low cost, simplicity of handling, the option of using either TE- or TM-polarised light for excitation, and the ability to deposit thin-films on both sides of the substrate^14^. In this case, unlike for optical fibers, the waveguide section is rectangular, but the phenomenon is quite similar to a multimode fiber. One of the modes guided in the coverslip is guided in the thin-film, and the phase matching of this mode with the modes guided in the coverslip is what causes an increase in the losses of these modes and, consequently, an absorption band in the transmission spectrum^5^. In addition, like for optical fibers, the transmission spectrum is generated by dividing the current signal with a reference signal obtained with a coverslip without thin film in the same setup, which avoids the generation of effects due to backward reflections. At the same time, without an automated system, the placement of the coverslip in the setup is not perfect and this leads to power losses in the baseline in the transmission spectrum that are not due to the deposition of the thin-film. This explains the differences in the base line of the transmission spectrum in the “Results” section.

Structures that generate LMRs in the optical spectrum can be used as sensors in a wide range of applications, such as detection of inorganic gases^15^, volatile organic compounds^16^, or voltage^17^. In fluids, they have been used to detect antibodies^18^, biomarkers^19^, or for monitoring electrochemical processes^20^. This success of LMR-based structures in sensors is largely due to their high sensitivity to refractive index^11,21^. Design rules for optimal sensitivity include optimisation of thin film thickness, thin film refractive index, and the refractive index of the surrounding medium^22^. These three parameters must be as high as possible to increase the sensitivity. However, by increasing these parameters, the LMR is shifted to longer wavelengths, ultimately making it impossible to track the LMR in the wavelength range of the spectrometer. It then becomes necessary to track a higher-order LMR, leading to a decrease in sensitivity, which is reduced for higher-order LMRs compared to lower-order LMRs. This lower sensitivity for higher order LMRs is due to the effective index of the mode guided in the thin film^6^; for higher order modes the effective index of the mode guided in the thin film is less sensitive to parameters such as thin film thickness and surrounding medium refractive index, which is related to the presence a thicker thin-film required for generating higher order LMRs. It has also been demonstrated that the surrounding medium refractive index is the most important of these three parameters. Consequently, to optimise sensitivity, the difference in refractive index must be minimised through the choice of the substrate^8^, the surrounding medium^23^, or both. Another interesting property is the capability of generating multiple LMRs in the optical spectrum. This property opens the path for obtaining multiple resonances in the same spectrum belonging to coatings with different thickness deposited on the same substrate.

However, each LMR corresponds to a different lossy mode order, which leads to quite different sensitivities that drastically decrease as a function of the mode order. Moreover, the separation between the LMRs is difficult to control because the wavelengths of the LMRs depend mainly on the three parameters that control the sensitivity, i.e., the refractive index, thickness of the thin-film, and the surrounding medium refractive index, which are fixed in each application the LMR generating structure is used.

This issue can be solved by exploiting two more properties of LMR-generating structures: the simplicity in tuning the position of the resonance, just by controlling the thickness of the coating^8^, and the capability of obtaining the LMR in a broad spectrum if the material satisfies the conditions for generating LMRs in that wavelength range^24^.

In the design proposed here, we gain control of the thin-film thickness in order generate multiple resonances in the transmission spectrum. To this purpose, a gradient in the thickness-based deposition method will be applied. This technique permits, with a single deposition, the generation of a structure on a planar waveguide that, subdivided in discrete sections, sets the basis for the translation of the concept of interdigitated electrodes, from the point of view of the structure, to the domain of photonic sensors.

## Results

### Generation of patterns on coverslips

**Figure 1:**
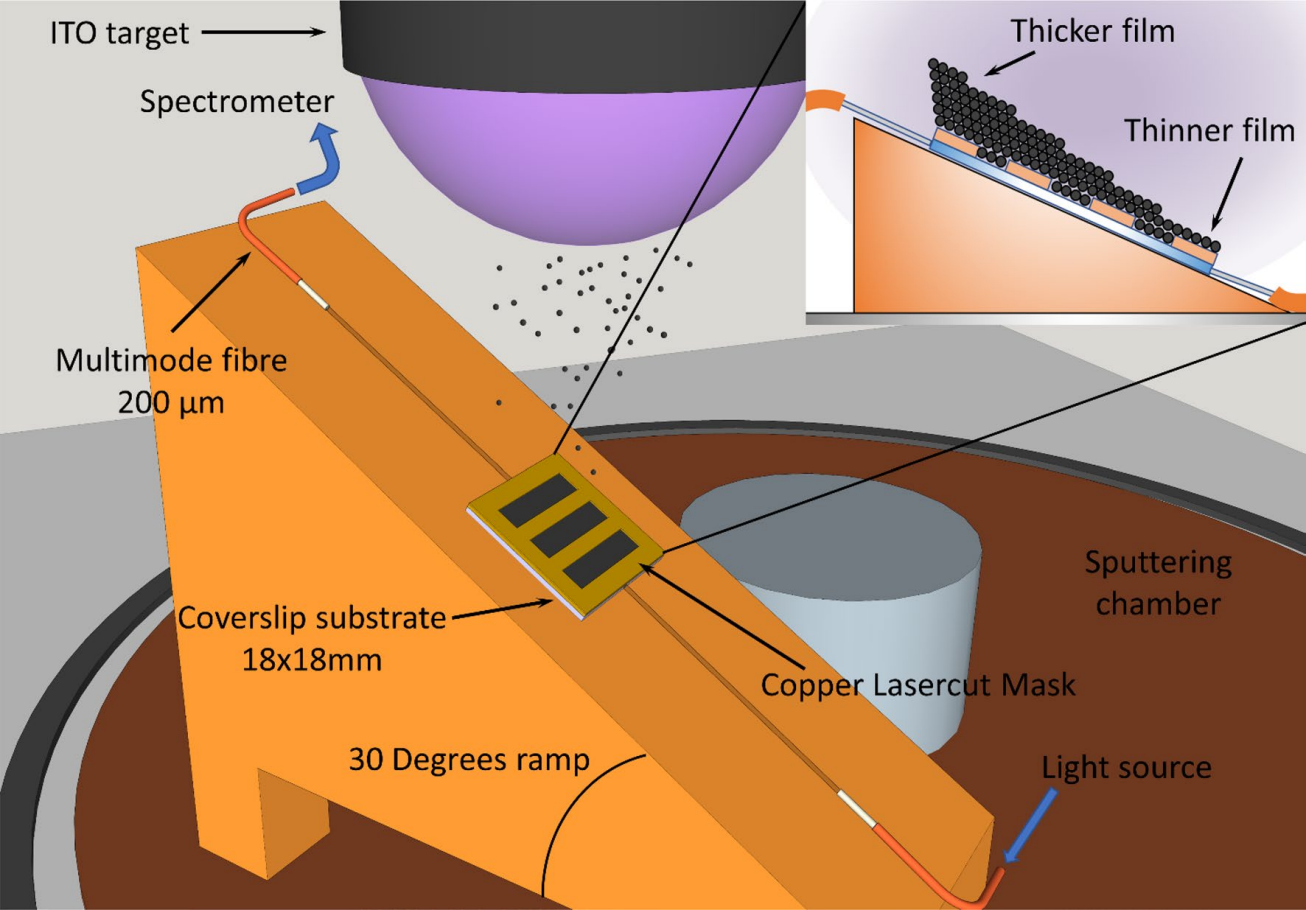
Deposition of a coating with a gradient in thickness. The waveguide, a simple coverslip for a microscope slide, was placed on a platform with a slope, which permitted creation of a non-uniform pattern on the surface of the coverslip**.**

The DC sputtering-based setup for deposition of an indium tin oxide (ITO) coating with a gradient in thickness is shown in Fig. 1. A platform with a slope was used for deposition of an indium tin oxide (ITO) coating with a gradient in thickness. More details on the conditions for the sputtering process are given in “Methods” section.

**Figure 2:**
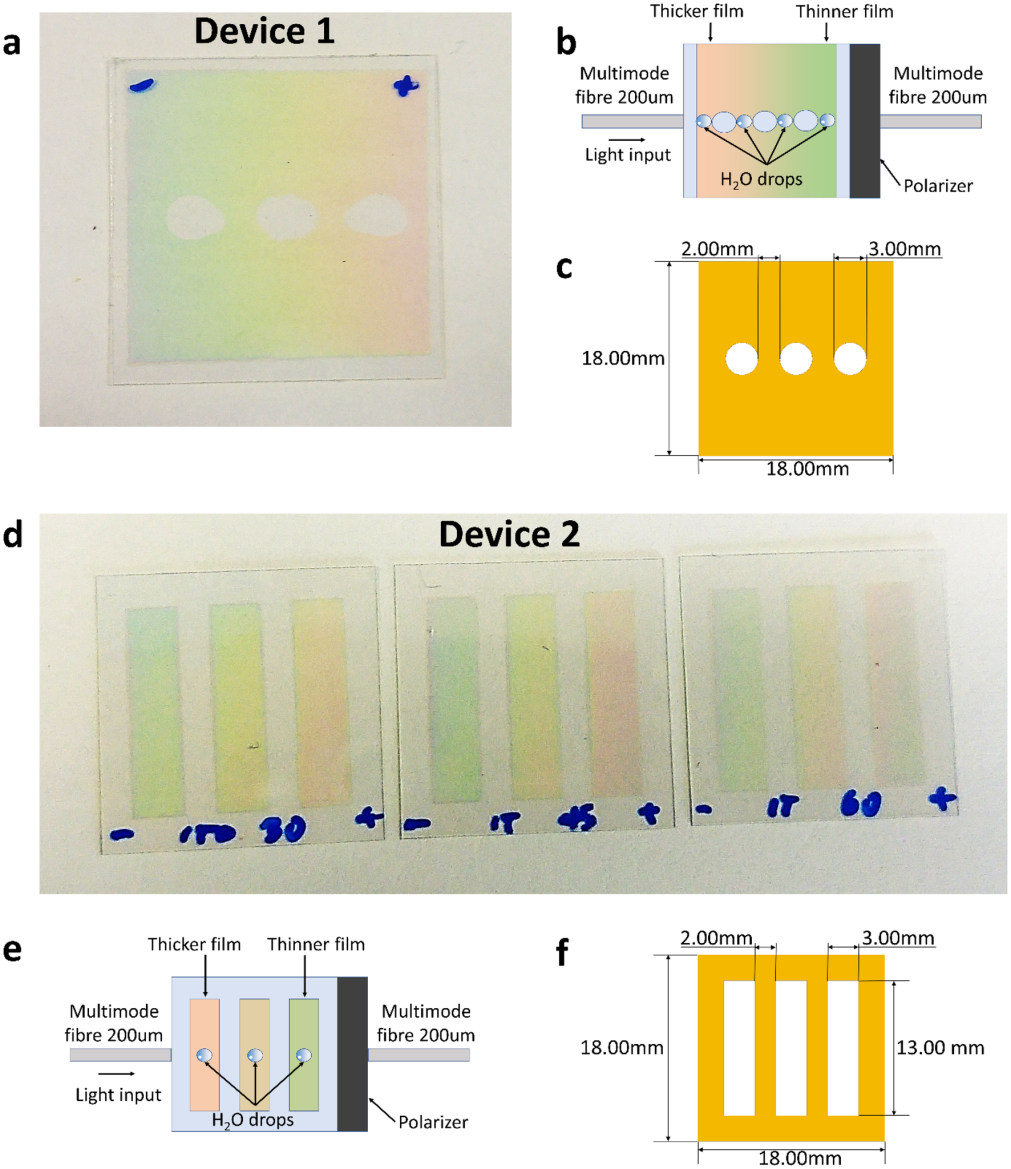
Samples deposited with and without a mask. **(a)** Device 1: experimental sample deposited with a gradient in thickness and segmented with HCl etching in different regions. **(b)** Details and dimensions of the mask used when removing the coating of the sample shown in **(a)** using HCl. **(c)** Schematic of the experiment on the sample shown in this figure: water drops were placed on top of each region deposited with different thickness. **(d)** Device 2: experimental sample deposited with a mask with platform of slope 30, 45, and 60, respectively. **(e)** Schematic of the experiment with water drops placed on top of the strips or electrodes in **(d)**. **(f)** Dimensions of the mask used for generating the strips or electrodes in **(d)**.

The setup has two variations. In the first option, henceforward Device 1, the coating is deposited directly on a coverslip and later patterns are made by etching the coating using a mask and pouring drops of hydrochloric acid (HCl) on the spots. This permitted isolation of different sections along one of the axes of the coverslips, which can be used for generating multiple LMRs in the optical spectrum. Figure 2a shows the ITO-coated coverslip with three circular regions etched using HCl and with the help of the mask whose design is depicted in Fig. 2b. This means that there are four deposited regions with different thicknesses, which can be verified by the different colour of the coating along the horizontal axis in Fig. 2a. Each of these four regions induces a different LMR in the transmission spectrum that will be shifted if, for instance, a drop of water is placed on top of each region, thus changing the surrounding medium refractive index (see Fig. 2c).

In the second variation, henceforward Device 2 (see Fig. 1), regions are directly generated during the deposition process by using a different mask. For this last process, three different angles (30º, 45º and 60º) were explored in order to verify the influence of this parameter on the generation of the LMRs. Figure 2d shows the patterns created with the three different angles, which again shows the interference colours in the horizontal axis indicating the gradient in thickness of the ITO coating. The dimensions of the mask used for the creation of the pattern are shown in Fig. 2e and more details are given in “Methods” section. Finally, Fig. 2f shows an experiment testing the wavelength shift of the LMRs induced by placing drops of water on top of each strip or electrode (analogous to an interdigital sensor).

### Controlling the position of multiple LMRs with different deposition patterns

Results are now shown for the two techniques proposed in “Results” for generation of multiple LMRs in the transmission spectrum. Both methods are based on the deposition of a coating with a gradient in thickness on a coverslip for a microscope glass slide. However, whereas in the first one, hydrochloric acid etching is applied for separating the different regions of the coating once deposited, for the second case a mask is used during the deposition process.

**Figure 3:**
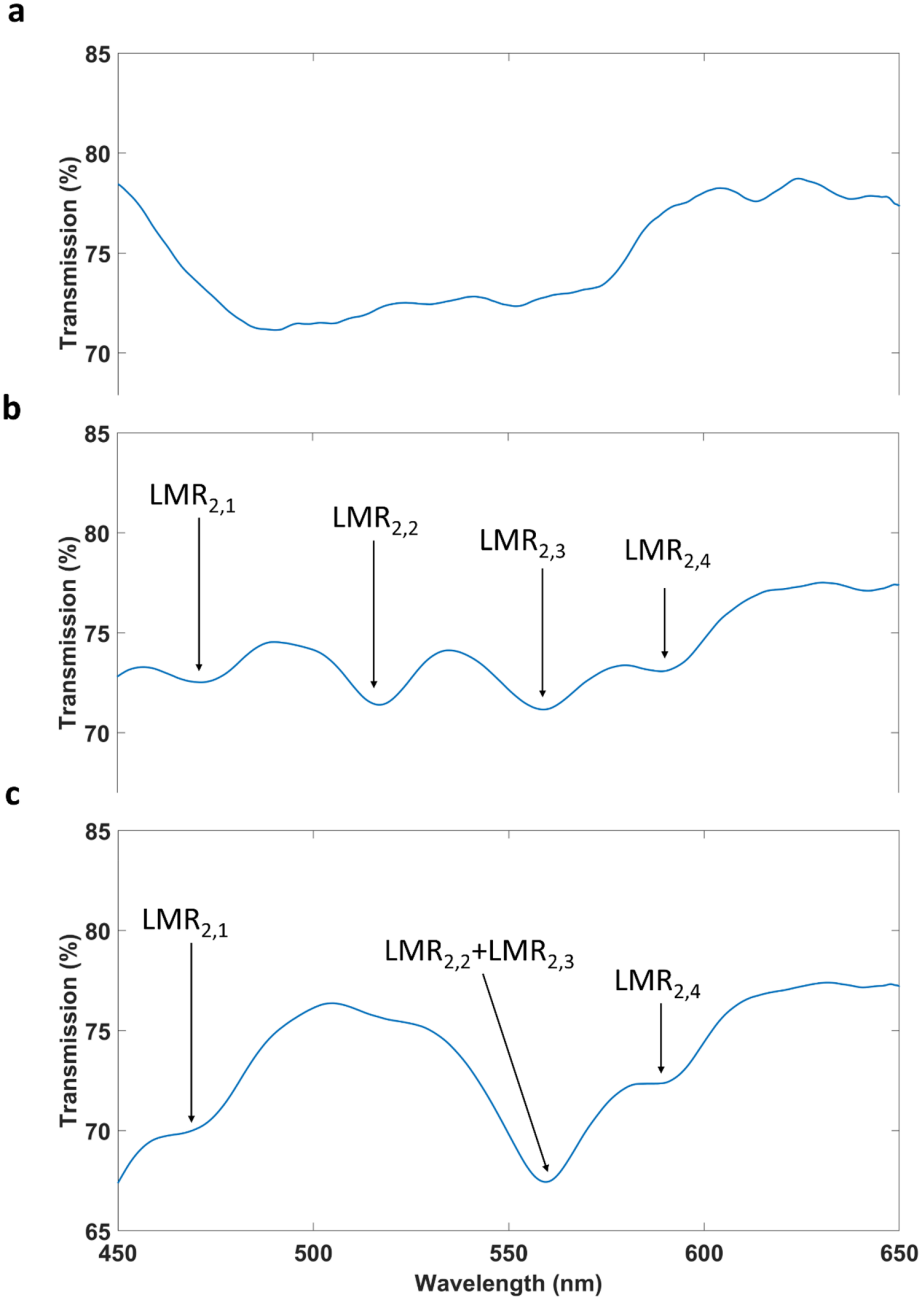
LMRs generated with device 1. HCl was used to separate four different regions of the coating: **(a)** LMRs when the all the four regions are surrounded by air (RI = 1); **(b)** LMRs when the all the four regions are surrounded by water (RI = 1.33); **(c)** LMRs when the all the regions are surrounded by air except for region 2, which is surrounded by water.

Figure 3a shows the TE-polarised spectra after the deposition of a thin film with a gradient in thickness, shown in Fig. 2a, when surrounded by air. The second LMR was selected because it is less sensitive than the first one (the first LMR shows a sensitivity around 1000 nm/RIU^25^, whereas the second one shows typically a fivefold decrease in sensitivity down to 200 nm/RIU^13^). This permits an easier tracking of the different resonances generated in the optical spectrum after the etching process with hydrochloric acid^8^. Therefore, the nomenclature to be followed will be LMR_2,X_, indicating the second LMR corresponding to the region X of the sensing platform. In Fig. 3b, the four resonances are observed, and in Fig. 3c, a drop of water was placed on top of one of the four regions of the coating (the region responsible for LMR_2,2_), generating a shift to longer wavelengths that coincides with the position of LMR_2,3_.

### Multiple LMR generation by mask-based deposition of thin films with gradient

**Figure 4:**
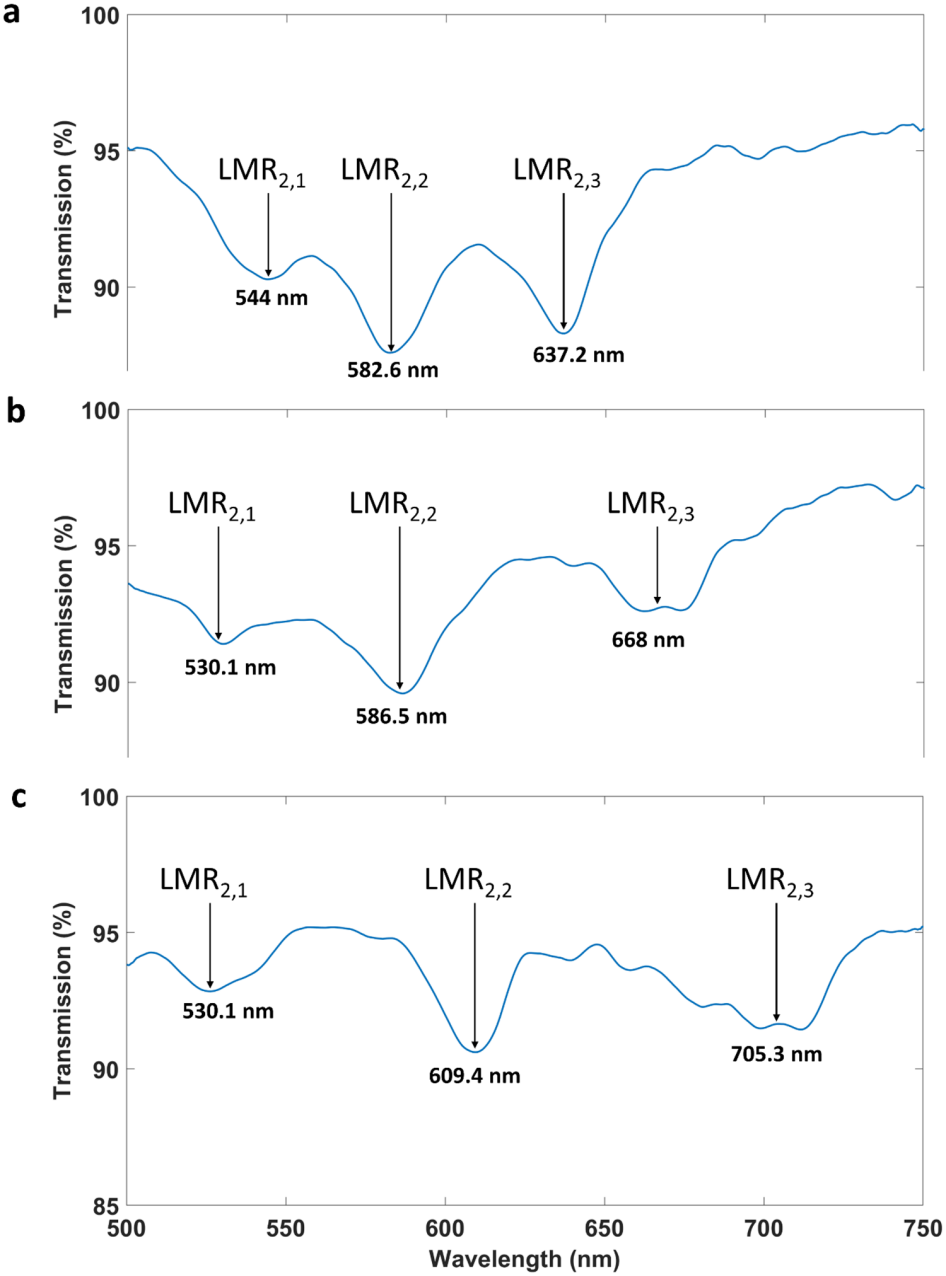
LMRs generated with device 2 for different deposition angles. A mask was used to separate three different regions during the coating process. Three different angles of deposition were analysed: **(a)** 30º, **(b)** 45º, and **(c)** 60º.

Figure 4 shows the three LMRs generated by the three deposited regions on the coverslip applied using a mask for separation during deposition. The angle evidently plays a role in the separation of the LMRs. A higher angle leads to an increase in the separation of the LMRs because the gradient in thickness is higher. This higher gradient in thickness means that, if comparing the three deposited regions, there is a higher contrast in the average value of the thickness in each region, and it is well known that the position of the LMR in the optical spectrum is related to the coating thickness^8^. Table 1 shows the wavelengths of the different LMRs. LMR_2,1_ and LMR_2,3_, the two extreme LMRs, are separated 93.2, 137.9, and 178.8 nm, respectively, for angles 30, 45, and 60, demonstrating that the separation can be increased by a factor of 2 by doubling the angle of deposition. However, when the angle is higher, the LMRs are broader and it is more difficult to extract the central wavelength. These values, 30, 45 and 60 were selected because they permit to compare and observe the difference in the separation of the LMRs when different angle of deposition is used. Indeed, for LMR_2,3_, obtained for angle 60, the peak shows oscillation, and effect that is also due to the fact that this LMR, located at a higher wavelength, is generated with a thicker coating deposited in the region of the substrate that is closer to the target. In sputtering deposition, when the target is closer to the substrate the deposition is less uniform, and effect that is exacerbated by the higher angle used in this case, 60 and which leads to a broader, oscillating peak. This would be solved by rotating the substrate or increasing the distance to the cathode.

**Table 1:** Wavelengths of the LMRs generated by coatings with a gradient in thickness induced by different deposition angles.

Deposition angle (degrees)	LMR_2,1_ (nm)	LMR_2,2_ (nm)	LMR_2,3_ (nm)
30	544	582.6	637.2
45	530.1	586.5	668
60	526.5	609.4	705.3

**Figure 5:**
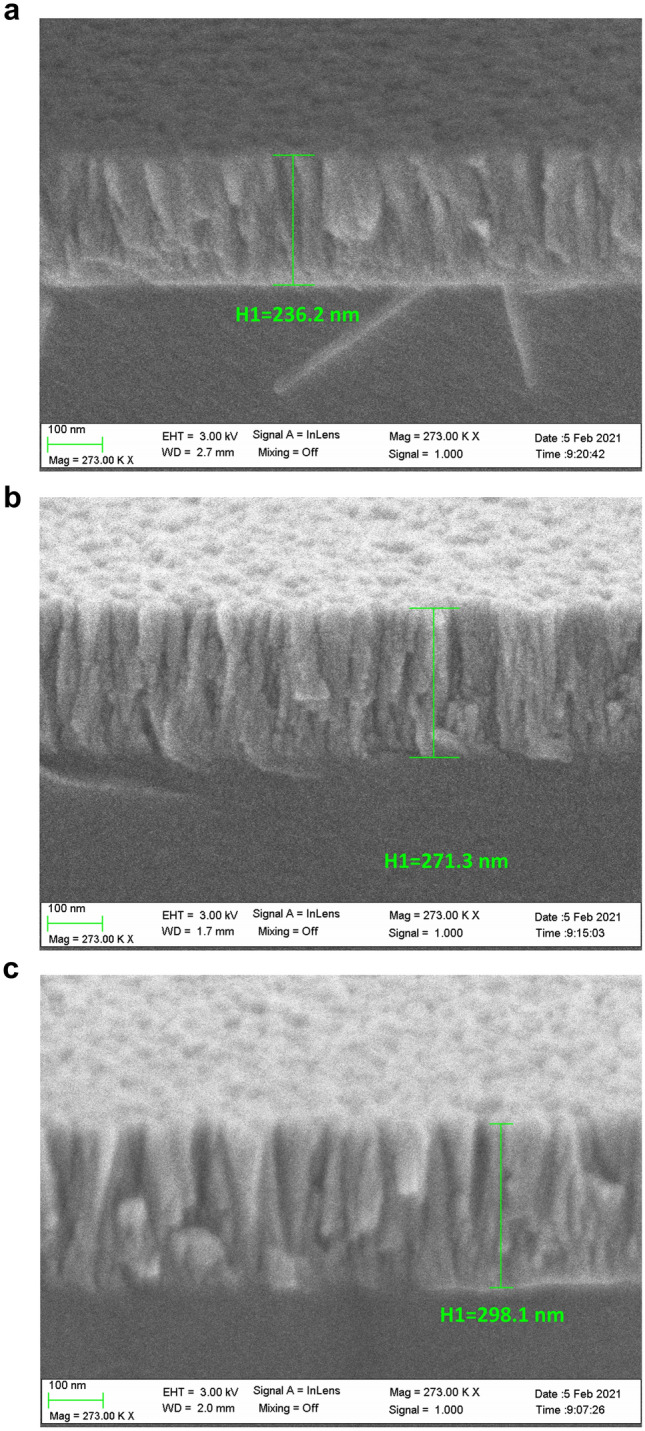
Scanning electron microscope (SEM) images for the sample deposited using a mask during the deposition process and a platform of angle 30º: **(a)** first electrode corresponding to the thinner region; **(b)** intermediate thickness; and **(c)** thicker region.

Samples were also characterised with a SEM microscope whose characteristics are shown in the "Methods" section. Figure 5 shows SEM images of the thickness in each of the three electrodes of the sample deposited with a mask during the deposition process in a platform of 30. A difference in thickness of 61.9 nm is observed when comparing the thicker and the thinner sample. The samples deposited in platforms of 45 and 60 were also analysed and the thickness difference was 77.2 and 91.2 nm, respectively. The SEM images are shown in Fig. S1 and S2 of the supplementary material.

**Figure 6:**
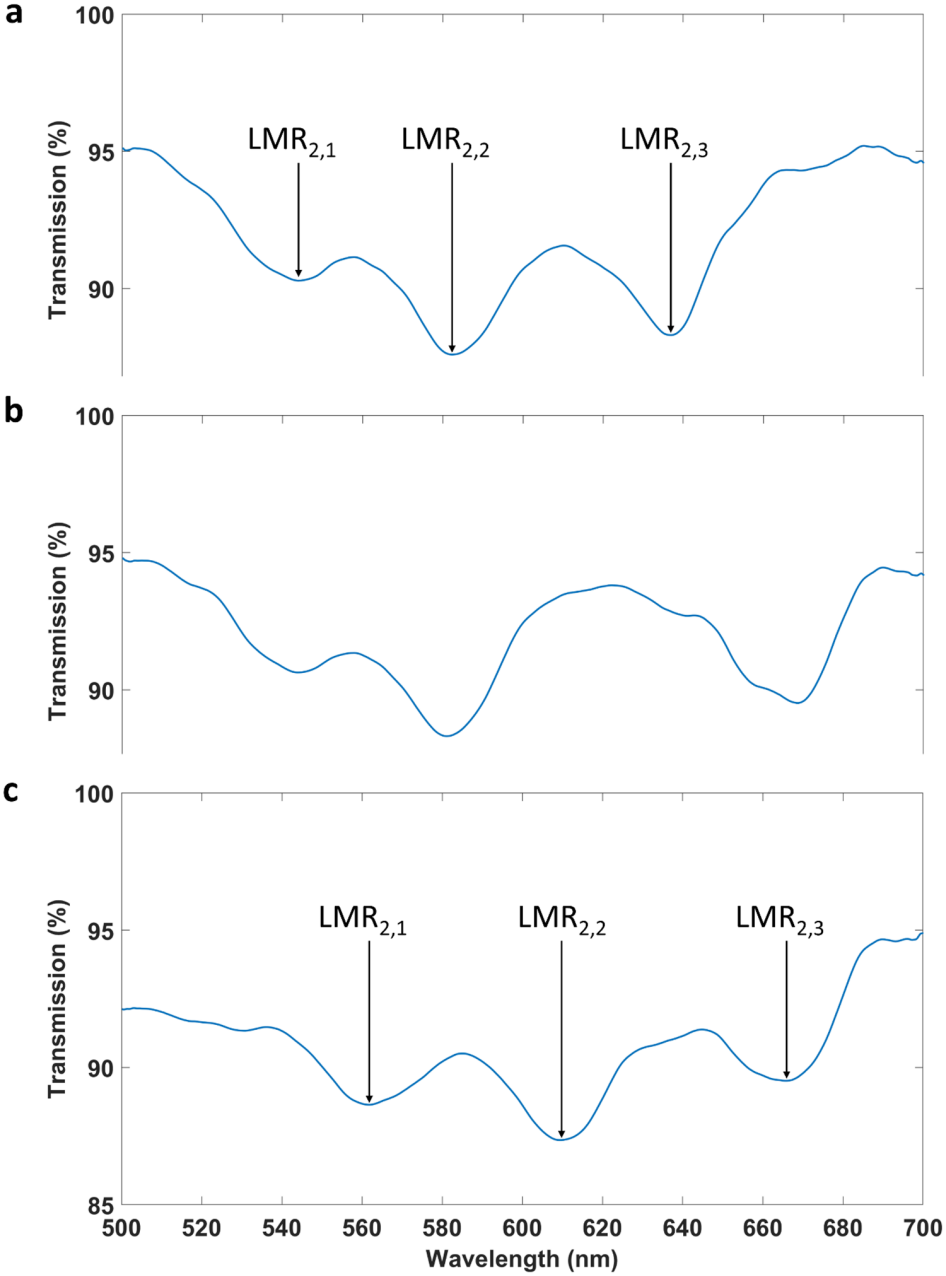
LMRs generated with device 2 (angle of deposition 30º) for different surrounding medium refractive indices. **(a)** LMRs when the all the three regions are surrounded by air (RI = 1); **(b)** LMRs when the all the regions are surrounded by air except for region 3, which is surrounded by water; **(c)** LMRs when the all the three regions are surrounded by water (RI = 1.33).

In view of the better resonances obtained with a platform of slope 30, this case was selected for the analysis performed for the structure studied without the mask, where the separation of the coated regions was generated by etching. Figure 6 shows the LMRs in air, when the region responsible for the generation of LMR_2,3_ is immersed in water and when all the regions are immersed in water. This demonstrates that the resonances are independent from each other.

**Figure 7:**
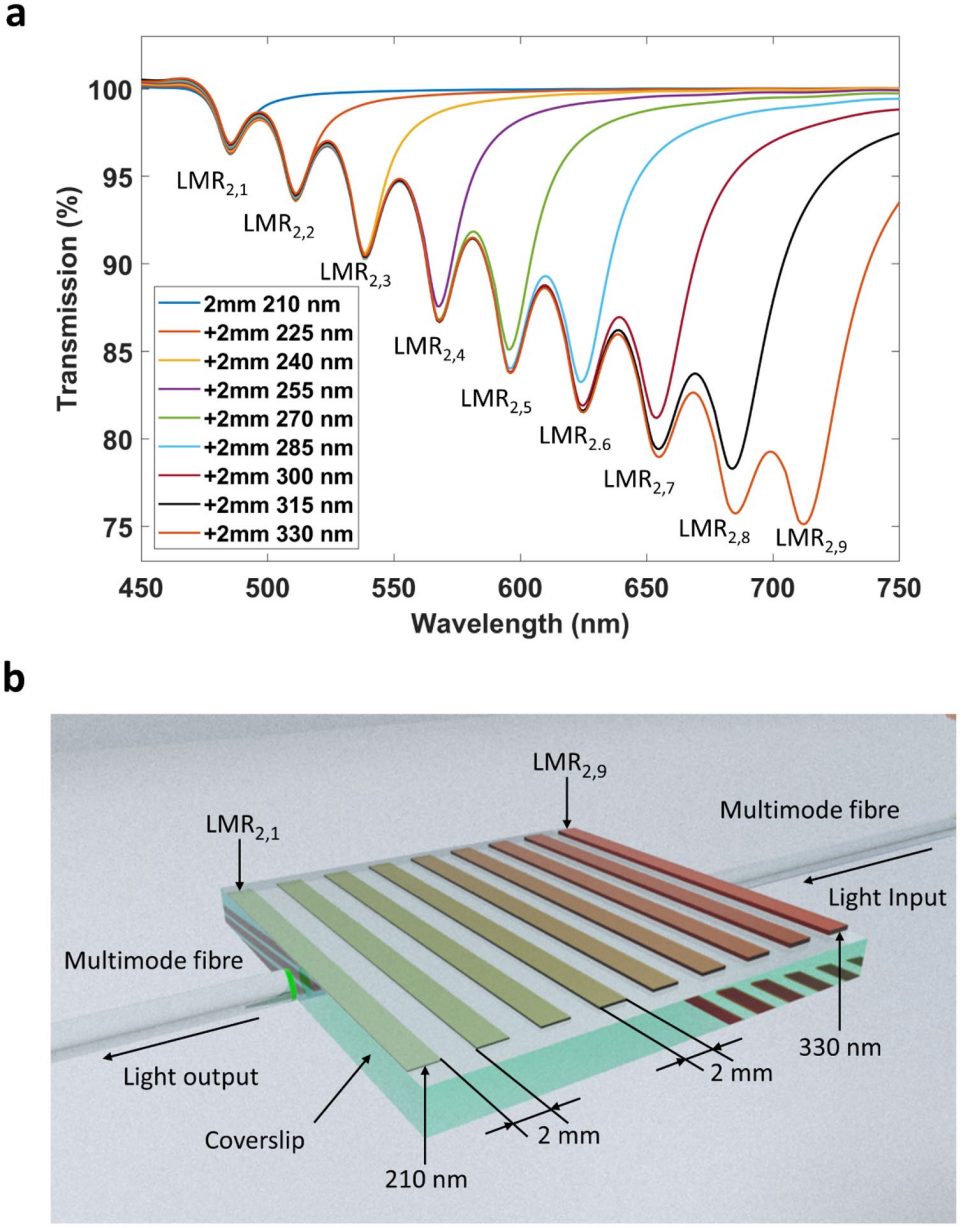
Array of 9 LMRs. **(a)** Simulation of the progressive addition of 2 mm electrodes, each one nanocoated to a different thickness, which permits generation of multiple LMRs in the transmission spectrum. The structure was surrounded by air (RI = 1). **(b)** Schematic representation of the array of 9 electrodes generating the corresponding 9 LMR-based sensors, where each one is identified with the resonance peaks of the graphic above.

Finally, in order to demonstrate the potential of the concept presented in this work, some simulations were performed with the commercial software package FimmWave, which has been successfully used for demonstrating that a gradient in thickness in a thin-film nanocoated optical fiber can induced a broadening in the LMR^26^. The ellipsometer model for ITO thin films simulated here can be found in Fig. S3, and the rest of the parameters used for the simulations are given in the "Methods" section). Figure 7a is a representation of an array of 9 LMR based sensors over a coverslip coated with 2 mm ITO electrodes of different thickness, ranging from 210 to 330 nm in steps of 15 nm. In Fig. 7b) the transmission spectra of the nine sensors are shown: the first one corresponds with a 2 mm section coated with 210 nm of ITO. The second one corresponds to the same 2-mm section coated with 210 nm with a 2-mm section coated with 225 nm of ITO, and so on until the last transmission spectrum, which corresponds to nine 2-mm sections with thicknesses ranging from 210 to 330 nm (the selection of 2-mm sections was based on the fact that the minimum length required to generate an LMR in the optical spectrum is 2 mm^26^). That is the sense for proposing in the structure of Fig. 7 a design with sections of length 2 mm. In any, case, for the sake of simplicity we have fabricated devices with section of around 4 mm in substrates of 18 × 18 mm. The selection of this area is based on the standard size of coverslips. The real part of effective index and the optical field intensity of these modes was also simulated and presented in Fig. S4 and S5 of the supplementary material.

## Discussion

The ability to isolate LMRs in the optical spectrum paves the way for the development of multiresonance platforms where multiple parameters can be detected. Here we present a simple approach based on deposition, on a coverslip for a microscope glass slide, of a nanocoating with a gradient in thickness. This permitted broadening of the LMR. Subsequently, an appropriate mask was used to isolate the LMRs by etching certain regions of the nanocoating, enabling the application of patterns with multiple sensor units on the surface of the substrate, in this case a simple coverslip. This permitted to obtain several separated LMRs for the broad LMR generated during the deposition. A second method consisted of generated the separated LMRs using a mask during the deposition, which leads to the generation of stripes on the surface of the coverslip separated from each other by non-deposited regions, a structure that resembles interdigitated electrodes, opening the path towards the extension of this concept to the domain of photonic sensors. The degrees of freedom in the proposed system are many. For instance, the angle of the platform used for the deposition of the coating with a gradient in thickness permits tuning of the separation of the LMRs (a higher or lower angle will lead to a higher or lower contrast of the regions deposited through the holes of the mask). Moreover, staircase or other cross-sectional profiles of the thin film can be generated using automated shifting masks. Here we use a single material, ITO, but it is possible to deposit different metallic oxides, or even materials that generate different phenomena, such as metals that induce a surface plasmon resonances (SPR) in the same structure. The interrogation of the sensor array was carried out all at once and perpendicular to the electrodes, but it could also be done longitudinally and individually for each of the electrodes. Taking into account the metallic nature of the deposited electrodes and the possibility of modifying their optical properties by applying voltage or current, the characteristics of the sensors can be modulated as a function of an applied electrical signal. In other words, the design possibilities with this concept are countless. Regarding the domains of application, as stated in the introduction, chemical sensors and biosensors require multiparameter sensing, whilst in the environmental domain or in smart cities it is also critical to detect multiple variables. The purpose of this work is to encourage the scientific community to advance on this topic.

## Methods

### Experimental setup for generating a thin film with a gradient in thickness

A DC sputtering machine (K675XD, Quorum Technologies, Ltd.) was used to generate a thin film with gradient thickness on a coverslip. The target for the deposition process was composed of indium tin oxide (ITO) with 57 mm in diameter, 3 mm in thickness and 99.99% purity, from ZhongNuo Advanced Material Technology. Deposition was performed under an argon partial pressure of 8 × 10^−2^ mbar and an intensity 150 mA. The substrates used for the deposition were coverslips from (RS, France), made from a conventional material, soda lime glass, with the dimensions 18 × 18 × 0.15 mm. The particularity in the setup of Fig. 1 is that, contrary to the standard procedure, the substrate was positioned with an angle related to the plane of the target surface, which permitted deposition of a different thickness in one of the axes of the coverslip. The mask was made of a 200 μm thick copper foil, cut on a laser machine in such a way that the holes created a pattern of three regions with differential thickness.

In order to monitor the optical spectrum during the experiments, a broadband source ASBN-W tungsten-halogen broad-band source (Spectral Products, Inc.) was connected to one end of a multimode optical fibre from (Ocean Optics, 200/225 μm core/cladding diameter). The other end was placed in front of one of the edges of a coverslip for a microscope slide acting as a planar waveguide. The output light of the coverslip was received by another multimode fibre (Ocean Optics, Inc.) connected to a USB2000 spectrometer (Ocean Optics, Inc.). This setup monitored wavelengths in the range 400–1000 nm.

### Characterisation of the samples

An ellipsometer (UVISEL, Horiba Scientific Thin Film Division) with a spectral range of 0.6–6.5 eV (190–2100 nm) was used to characterise the refractive index and the extinction coefficient of the thin film and the gradient in thickness of the samples. To corroborate the results of the ellipsometer, we used a field-emission scanning electron microscope (UltraPlus FESEM, Carl Zeiss, Inc.) with an in-lens detector at 3 kV and an aperture diameter of 30 μm. Both measurements give an estimate of coating thickness. All values were used in the simulations are given in the “Results” section and in the supplementary material.

### Simulations

Simulations were performed to obtain a deeper knowledge on the phenomenon. The propagation through the coverslip waveguide was obtained with FimmProp, an integrated module of FimmWave. The finite difference method (FDM) with the Quasi 2D option was used to calculate the modes and fields in the cross-section of the waveguide for a total number of 40 modes. In addition, a Gaussian source with a 100 µm half width at half maximum was used according to the 200 µm multimode fibre used in the experiments for exciting the planar waveguide. The dispersion curves of the coverslip, made of soda lime glass, and the substrate that supported coverslip, made of PMMA, were also considered in the simulations^27,28^. Here it is important to indicate that soda lime glass presents a higher refractive index than the PMMA, which permits to use it as a waveguide for the transmission of light. In addition, absorption losses of soda lime glass are negligible in the wavelength range analysed, with an extinction coefficient lower of the order of 10^–5^ and 10^–6^^27^.

## Supplementary Information

Supplementary Information.
