# Supplementary material for: Interdigital concept in photonic sensors based on an array of lossy mode resonances
Source: Sci Rep. 2021 Jun 24;11:13228. doi: 10.1038/s41598-021-92765-0 (PMC8225812; doi:10.1038/s41598-021-92765-0)
Supplement: Supplementary file 1 — Supplementary Information. [file 41598_2021_92765_MOESM1_ESM.docx]

SUPPORTING INFORMATION

Array of lossy mode resonances generated by a nanocoating with a gradient in thickness: towards the interdigital concept in photonic sensors

Ismel Dominguez^1,2^, Ignacio Del Villar^1,3^, Omar Fuentes^1,2^, Jesus M. Corres^3^, Ignacio R. Matias^1^*

^1^Institute of Smart Cities, Public University of Navarre, 31006 Pamplona, Spain

^2^Department of Telecommunications and Electronics, Pinar del Río University, Pinar del Río CP 20100, Cuba

^3^Department of Electrical and Electronic Engineering, Public University of Navarre, 31006 Pamplona, Spain

[*natxo@unavarra.es](mailto:*natxo@unavarra.es)

SEM Microscope images for samples deposited with platform of slope 45 and 60º

Microscope images are shown in Fig. 5 for the thickness in each of the three strips of the sample deposited with a mask in a platform of 30º. Here, the same analysis is performed for the samples deposited with a platform of 45º (Fig. S1) and 60º (Fig. S2). The images were obtained with a field emission scanning electron microscope (UltraPlus, FESEM, Carl Zeiss, Inc.) with an in-lens detector at 3 kV and an aperture diameter of 30 μm. The main conclusion is that the increase in the angle of deposition leads to a higher gradient in the nanocoating, which agrees well with the higher separation of the LMRs obtained for higher angles in Fig. 4.

Ellipsometer measurements

The refractive index and the extinction coefficient of the ITO coating were characterized with an ellipsometer (UVISEL 2, Horiba), with spectral range of 0.6ꟷ6.5 eV (190ꟷ2100 nm), an angle of incidence of 70°, a spot size of 1 mm, and software DeltaPsi2TM (from Horiba Scientific Thin Film Division). The results are shown in Fig. S3, where it can be observed that the ITO coating meets the conditions for LMR generation over the wavelength range analysed (400ꟷ700 nm). For this wavelength range the real part of the thin film (ITO) permittivity is positive and higher in magnitude than both its own imaginary part and the material surrounding the thin film^1,2^. Mathematical expressions defining these conditions are given in Table 1, under the assumption that the outer medium presents a lower refractive index than the substrate.

**Figure S1 | Scanning electron microscope (SEM) images for the sample deposited with mask and a platform of angle 45º**: (a) thinner region; (b) intermediate thickness; (c) thicker region.

**Figure S2 | Scanning electron microscope (SEM) images for the sample deposited with mask and a platform of angle 60º**: (a) thinner region; (b) intermediate thickness; (c) thicker region.

**Table S1.** Conditions for generation of SPRs and LMRs ^a^

**LMR**

^a^ ε_1_, ε_2_, n_1_, n_2_, k_1_, and k_2_ represent the permittivity, refractive index, and extinction coefficient for the substrate (1) and the thin film (2) respectively

**SPR**

**LMR**

**Figure S3 | Refractive index (n) and extinction coefficient (k) of the ITO nanocoating.** The results indicate that the conditions for LMR generation are met in the wavelength range analysed (400ꟷ700 nm). Above 1600 nm, SPR generation conditions are met.

Simulations

Fig. 7 shows the simulated transmission spectra corresponding to the progressive addition of 2 mm segments deposited with a thickness ranging from 210 to 330 nm in 15 nm steps. FimmProp, an integrated module of Fimmwave, was used to generate these simulations. The parameters for the simulation are detailed in Methods section. Here, the real part of effective index of the modes is presented in Fig. S4. Forty modes were simulated, and the fundamental mode, in read, experiences a transition to guidance in the ITO thin film at the wavelength of the corresponding resonance in Fig. 7. There are 9 red lines because Fig. S4 shows the effective index of the modes for the 9 different thicknesses analysed in the simulations, and the transition occurs at a different wavelength depending on the thin-film thickness.

Additionally, the modal field of this fundamental mode guided in the coverslip was calculated for three cases: 210, 255, and 330 nm (Fig. S5). In all cases, transition to guidance in the ITO coating at different wavelengths can be observed. For the 210 nm case the transition is occurs at a wavelength shorter than 255 nm, whereas at 255 nm the transition occurs at a wavelength shorter than the 330 nm, as expected in lossy mode resonance-based sensors ^2^.

**Figure S4 | Real part of the effective index of the 40 modes analysed in Fig. 6.** One of the modes (red colour) experiences a transition to guidance in the ITO thin film. This analysis was performed for thicknesses ranging from 210 to 330 nm in steps of 15 nm and agreement is found between the transition wavelength and the position of the resonances in Fig. 7.

**Figure S5 | Optical field intensity distribution of TE_1_ mode in the cross-section of a coverslip waveguide coated with 210 nm (left column), 255 nm (central column) and 330 nm (right column).**

References

1.	Yang, F. & Sambles, J. R. Determination of the optical permittivity and thickness of absorbing films using long range modes. *J. Mod. Opt.* **44**, 1155–1164 (1997).

2.	Del Villar, I. *et al.* Optical sensors based on lossy-mode resonances. *Sensors Actuators, B Chem.* **240**, 174–185 (2017).
